# A Multi-Clustering Algorithm to Solve Driving Cycle Prediction Problems Based on Unbalanced Data Sets: A Chinese Case Study

**DOI:** 10.3390/s20092448

**Published:** 2020-04-25

**Authors:** Yuewei Wu, Wutong Zhang, Long Zhang, Yuanyuan Qiao, Jie Yang, Cheng Cheng

**Affiliations:** School of Information and Communication Engineering, Beijing University of Posts and Telecommunications, Beijing 100876, China; zhangwutong@bupt.edu.cn (W.Z.); 13269593383@bupt.edu.cn (L.Z.); yyqiao@bupt.edu.cn (Y.Q.); janeyang@bupt.edu.cn (J.Y.); trcheng@bupt.edu.cn (C.C.)

**Keywords:** unbalanced data, driving cycle, multi-clustering algorithm, stacking algorithm

## Abstract

Vehicle evaluation parameters, which are increasingly of concern for governments and consumers, quantify performance indicators, such as vehicle performance, emissions, and driving experience to help guide consumers in purchasing cars. While past approaches for driving cycle prediction have been proven effective and used in many countries, these algorithms are difficult to use in China with its complex traffic environment and increasingly high frequency of traffic jams. Meanwhile, we found that the vehicle dataset used by the driving cycle prediction problem is usually unbalanced in real cases, which means that there are more medium and high speed samples and very few samples at low and ultra-high speeds. If the ordinary clustering algorithm is directly applied to the unbalanced data, it will have a huge impact on the performance to build driving cycle maps, and the parameters of the map will deviate considerable from actual ones. In order to address these issues, this paper propose a novel driving cycle map algorithm framework based on an ensemble learning method named multi-clustering algorithm, to improve the performance of traditional clustering algorithms on unbalanced data sets. It is noteworthy that our model framework can be easily extended to other complicated structure areas due to its flexible modular design and parameter configuration. Finally, we tested our method based on actual traffic data generated in Fujian Province in China. The results prove the multi-clustering algorithm has excellent performance on our dataset.

## 1. Introduction

Car parameters are an important indicator to guide consumers in purchasing cars, and they quantify users’ driving experience as comparable figures. However, the direct use of other countries’ test standards will lead to some car parameters that cannot accurately reflect the actual driving experience of consumers in China’s automotive test projects, which can cause the information to be false or misleading and thus lead to confusion among consumers. At present, developed countries such as Europe, the United States, and Japan generally accept driving cycle standards and energy consumption/emission certifications that are adapted to their respective driving cycles. Due to the vast territory and complicated structure area, more customized algorithms are required for driving cycle prediction in China.

**Figure 1:**
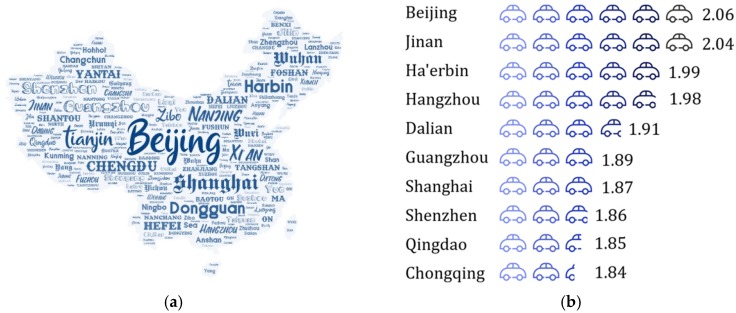
Basic situation of vehicle use. (**a**) China’s urban area distribution (name location does not represent actual city location, and text size represents the number of cars in each city); (**b**) Congestion delay index (2016).

Car fuel consumption is one of the indicators of most concern for car buyers. However, in the past, with the New European Driving Cycle (NEDC), published in Europe for energy consumption/emission evaluation and widely used in China, it is difficult to accurately illustrate the actual performance of cars in the various regions of China. Thus, the gap between the manufacturer’s published data and the actual test data of the users is extremely obvious, which seriously decreases the overall buying experience of users. The reason for the above result is China’s geographical location and economic development are vastly different from those in Europe, not only in many various car types but also in the complicated terrain of China. There are obvious three-level ladders in geographical trends and the economic level is high in the west and low in the east. The number of cars in developed cities is large but the urban area is generally small, as shown in Figure 1a. Therefore, the congestion delay index [1,2] is higher, as shown in Figure 1b. At the same time, the average elevation in Tibet, Yunnan and other places is very high, and there are problems, such as insufficient gasoline combustion. The above various reasons explain why the NEDC driving cycle evaluation system proposed by Europe cannot reflect the actual performance of cars in China.

In recent years, the rise of big data and artificial intelligence has provided a strong theoretical foundation for solving driving cycle prediction problems with deep personalization, and computer advancements have provided the necessary computing power.

In the driving cycle prediction research field, the predecessors were based on K-means clustering algorithms for unsupervised clustering analysis. K-means is a partitioned clustering technique that is widely known for its low computational complexity. Representative algorithms include hard k-means and fuzzy k-means, but these approaches always suffer from bad performance and are not suitable for skewed data distributions (i.e., unbalanced data). Even if the input data has different cluster sizes, they usually produce clusters of relatively uniform size, which is called "uniform effect" [3]. As we know intuitively, during normal vehicle driving, people usually keep driving at a medium speed (10–90 km/h). There are relatively few cases of low-speed driving (0–10 km/h) and ultra-high-speed driving (>90 km/h). Therefore, in a normal vehicle driving data set, the amount of data is obviously tilted toward the medium speed section. Therefore, the “uniform effect” largely affects the driving cycle prediction results, resulting in a large gap between the driving cycle map parameters and the actual vehicle driving experience.

To address the above limitations, we draw inspiration from ensemble learning to design the multi-clustering algorithm, a novel unsupervised learning algorithm. Usually, boosting, bagging and stacking are three effective algorithms used in the field of ensemble learning. This method is considered to have good performance in the processing of unbalanced data sets [4]. In the supervised learning algorithms of machine learning, the goal setting for the stable model that performs well in all aspects is often so ideal that it’s hard to achieve, and sometimes we can only get multiple models with preferences (weakly supervised models perform better in some respects). Ensemble learning combines multiple weak monitoring models in order to get a better and more comprehensive strong monitoring model. The underlying idea of ensemble learning is that even if a weak classifier gets a prediction wrong, other weak classifier can correct the error. In this paper, our main contributions are summarized as follows:

- A multi-clustering algorithm framework is proposed based on further improvement of the stacking algorithm framework. The algorithm framework integrates k-means-type, K-means++, DBSCAN and other clustering algorithms, and uses stacking to strengthen each individual model. Multi-clustering can solve potential vulnerabilities such as data imbalances and random initial states. We performed multiple experiments on the matching order and crossover method of the algorithm to obtain the best results. Through experiments, we have verified that our proposed multi-clustering framework performs well in the data set and offers a relatively high improvement over other algorithm.
- We use T-SNE algorithm instead of the principal component analysis (PCA) algorithm for data dimensionality reduction. In the field of traditional driving cycle map research, the PCA method is used for feature extraction and dimensionality reduction. This approach creates the problem of unclear division between categories. Therefore, we select the T-SNE algorithm instead of the PCA algorithm to reduce the dimensionality of high-dimensional features. After a comparison between the two different dimension reduction algorithms, it can be clearly seen from the experimental results that T-SNE performs better given the characteristics of our working data set.

The remainder of this paper is organized as follows: In Section 2, we investigate the methods used by predecessors in the field of driving cycle prediction and compare them with our research. In Section 3, we first introduce the overall flow of the experiments, and then detail the theoretical derivation and actual implementation of the basic algorithm used in the experiments. In Section 4, we describe the proposed multi-clustering algorithm, show the internal structure of the algorithm, and theoretically explain and deduct our proposed algorithm. In Section 5, we elaborate on the implementation of the actual experiments and a comparison of the effects of each algorithm with the multi-clustering algorithm. Furthermore, we conduct a detailed analysis of the experimental results in Section 6. Finally, we give the conclusions in Section 7.

## 2. Related Work

### 2.1. Unbalanced Datasets

In life, questions about the classification of unbalanced data sets are ubiquitous. For example, pathological analysis, traffic accident analysis, crime analysis, and so on. Predecessors have reported many achievements in the field of unbalanced dataset research [4,5,6,7].

Some researchers use sampling methods to balance data sets. Chawla et al. proposed the oversampling data processing method SMOTE [8]. Tahir et al. handled data imbalances by down sampling [9]. Later, Wang et al. proposed a problem that cannot be solved by simply using up sampling or down sampling, and tried to balance the dataset by using multiple sampling [10].

With the advancement of technology, some works have proposed a new loss function that calculates the loss of positive and negative classes separately, making the machine more sensitive to small classes [11]. To better measure losses, Khan et al. proposed a dynamic loss function. The parameters of the loss function are constantly modified with the iteration of the training to obtain better prediction results [12].

At the same time, some scholars are studying the problem of unbalanced data in the fields of unsupervised learning and ensemble learning [4]. The most widely used ensemble learning algorithms are AdaBoost [13] and bagging [14] whose applications in several classification problems have led to significant improvements [15]. At the same time, many scholars have combined data preprocessing and ensemble learning to propose algorithms such as SMOTEBoost [16], RUSBoost [17], SMOTEBagging [18] and EasyEnsemble [19].

### 2.2. Driving Cycle Prediction

Many scholars have conducted research in the field of driving cycle prediction [20,21,22]. They have proposed different prediction methods from the perspectives of engineering and mathematics. Huang et al. using kinematic segment classification to establish a basic driving cycle database including 4704 different transmission matrices. Based on the inter-station driving characteristic equations and the basic driving cycle database, a driving cycle prediction model was developed, generating drive cycles by the iterative Markov chain for assigned bus lines [23]. Chen et al. proposed an online correction algorithm based on a backup control strategy and a fuzzy logic controller [24]. Huang et al. proposes an intelligent multifeatured statistical approach to automatically discriminate the driving conditions of the hybrid electric vehicles (HEVs) [25]. Chinese researchers have also discussed the differences in vehicle driving characteristics between China and Europe and the United States, and have studied the roads in China. Wang et al. used 11 driving parameters to develop a formal cycle for China’s national conditions, and compared it with the standards proposed by Europe and the United States [26]. Li et al. used principal components analysis and cluster analysis of the features of driving speed, idling, maximum speed, acceleration and travel mileage, as well as their distribution features in three typical cities in China [20].

While the above approaches for driving cycle prediction have been proven effective and used in many countries, these algorithms are difficult to achieve the effect in china with complex traffic environment and increasingly high traffic jams. Meanwhile, we found the unbalanced data will have a huge impact on the performance when building driving cycle maps, and the car parameters generated by above algorithms deviate quite a lot from actual ones. In order to address the above limitations, this paper proposes a new algorithm framework for the driving cycle prediction problem from the perspective of machine learning and unbalanced data analysis. An experimental study was conducted in a Fujian (China) vehicle data set, and a driving cycle was proposed for Fujian. After our comparative analysis, we found that this problem has been studied from a fresh perspective and has a good performance. The multi-clustering algorithm is simple and easy to implement, and it is suitable for migration to different regions for practical applications, especially in China, where the terrain is complex and the urban development gap is large.

## 3. Preliminary

In this section, we firstly briefly introduce the general situation, classification and basic processing of the driving cycle map generation. Then, we describe in detail how to build the working condition map of our experiments and provide technical support for the subsequent experiments. At the same time, we also carried out theoretical derivation and verification of the algorithm used in the experiments, and this theoretical analysis proved the correctness of the subsequent experiments.

Driving cycles are often used to determine a vehicle’s 100-kilometer fuel consumption, pollutant emissions, and for the development, evaluation, or evaluation of traffic risks of new vehicles. It is a core technology in the automotive-related field.

### 3.1. Brief Description of the Driving Cycle

Vehicle driving cycles are also known as car operating cycles. The refer to the characteristics of a certain type of vehicle (such as light vehicles, buses, heavy vehicles, etc.) under certain circumstances. They are usually displayed in the form of a speed-time curve (usually the total time is 1800 seconds, but there is no standard limit). Because the vehicle driving cycle must reflect the actual running state of the car, we usually need to collect driving data in an actual environment with sensors and use various mathematical, physical, computer methods to process, screen and analyze it.

Driving cycle reflect the kinematics of road driving, which is an important and common basic technology for the automotive industry. As the main benchmark for the calibration and optimization of various performance indicators of automobiles, it is also the basis of vehicle energy consumption/ emission test methods and limit standards. For example, it can be used to determine vehicle fuel consumption and pollutant emissions per 100 km, to determine whether the car really saves energy as advertised. In addition, as a core technology of automobile-related fields, it is also widely used in the development of new cars, performance evaluation, and assessments of traffic risk [27]. 

### 3.2. Driving Cycle Classification

In the field of driving cycle research, the maps are generally divided into two categories: transient conditions and modal conditions, according to the characteristics of the speed-time curve.

FTP75, proposed by the United States based on FTP72 in 1975, is a typical transient condition cycle. Such working condition maps are directly constructed from real road traffic data, with the advantage of easily reflecting the various driving behaviors, such as rapid acceleration and emergency stops, but they cannot fully illustrate the detailed information of driving behaviors during the test, because the driving behavior is complicated and in fact there are many details.

The NEDC driving cycle proposed by Europe has all the basic characteristics of modal conditions. It synthesizes a representative working condition map by performing modal analysis on real traffic data. The modal condition map does not have too many sudden changes in acceleration, and each state has a longer holding time, which can be better used for scenes such as factory tests of the automobile. In general, the velocity-time image of the driving cycle map is a continuous smooth curve while the velocity-time diagram of the modal map is a line graph.

### 3.3. Method for Constructing Transient Map

**Figure 2:**
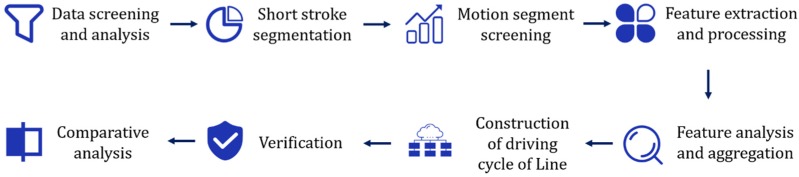
Transient driving cycle map construction process.

When constructing the transient driving cycle map, we mainly analyze and construct it according to the flow shown in Figure 2, and use the short stroke method for data analysis [28]. The short stroke method is a common method for constructing driving conditions. The short stroke, also known as the kinematics segment, refers to the range of speeds between the start of the idling state and the start of the next idle state, including an idle period and a period of exercise. The method first divides the data into multiple kinematic segments. Then the computer method is used to explore the correlation between each motion segment, to select the most representative short-stroke segment. Finally, according to the 36 features and data ratios, such as speed, acceleration, displacement, and load ratio, the short-stroke segments are stitched together to obtain the final transient working condition diagram.

#### 3.3.1. Build Step

- First, we analyze the data given, study the data content, quality, analyze the causes of noise and find ways to denoise the data. The data set is processed into a high quality, low error state by means of completion, screening and the like.
- Secondly, find the appropriate way and rule to divide the kinematics after the data screening is completed. Compare the different division methods, and choose the division method with the most practical application meaning and the most analytical competitiveness for later use.
- Thirdly, we extract and process all the features in the data set, merge the redundant information, delete the invalid information, and finally get 36 features. Then, through the T-SNE algorithm, the features are reduced in dimension to facilitate subsequent calculations.
- Fourthly, we first try to cluster the group feature data using the K-means algorithm. Several improved algorithms are proposed for the problem of K-means algorithm. In order to concentrate the advantages of several algorithms and reduce the model bias, a multi-clustering algorithm based on unsupervised integrated learning is proposed. Then the clustering result is converted into a condition map by Markov chain method.
- Finally, we compare and verify the correctness of the proposed driving cycle diagram and compare it with the working condition diagram proposed by the predecessors. At the same time, the effect of our proposed multi-clustering algorithm framework on traditional algorithms is improved, and the final analysis leads to the conclusions.

#### 3.3.2. Drop Feature Dimension

In the field of driving cycle prediction research, most of the existing papers use the principal component analysis (PCA) algorithm [29] to reduce the dimensionality of high-dimensional features.

The main job of PCA is to sequentially search for a set of mutually orthogonal axes from the original high-dimensional space. The first axis created is selected from the one with the largest variance in the original data. The second coordinate axis selects the direction in which the original data variance is the largest in the plane orthogonal to the first coordinate axis. The third coordinate axis selects one direction in which the original data variance is the largest in a plane orthogonal to the first and second coordinate axes, and iterates according to this step until the k (target dimension) coordinate axes are selected. In the coordinate axis found in this way, the top k coordinate axes with the largest variance are selected, and the variances of the remaining coordinate axes are very small and can be ignored. Therefore, PCA achieves data feature dimensionality reduction by ignoring the dimension with very small variance [30].

PCA is a linear dimensionality reduction algorithm. However, it cannot explain the complex polynomial relationship between features. Therefore, we use T-distributed stochastic neighbor embedding (T-SNE) algorithm to reduce the dimension of the 36 features data.

The T-SNE algorithm is an improved version of the stochastic neighbor embedding (SNE) algorithm. It is based on the probability distribution of random walks on the neighborhood graph to find the structure within the data. The dimensionality reduction effect is excellent, but the calculation amount is large, and the system resources are high [31,32]. Our experimental team has cluster computing resources that are enough to support the T-SNE algorithm, so we use the more effective T-SNE algorithm for dimensionality reduction. The T-SNE algorithm maps data points to probability distributions by affine transformation. The main process is divided into two parts. Firstly, construct a probability distribution between high-dimensional objects. This makes similar objects more likely to be selected and dissimilar objects less likely to be selected. Secondly, the probability distribution of these points is constructed in a low dimensional space such that the two probability distributions are as similar as possible. From an algorithm perspective, the overall T-SNE algorithm is as follows:

- The first step: Using the SNE algorithm, starting by converting the high-dimensional Euclidean distance between data points into a conditional probability of similarity, the conditional probability between the calculated data points $x_{i}$ and $x_{j}$ is as shown in Equation (1):
  (1) $$p_{j|i}=\frac{\exp\left(\frac{-{\left|\left|x_{i}-x_{j}\right|\right|}^{2}}{2\sigma_{i}^{2}}\right)}{\sum_{k\neq i}\exp\left(\frac{-{\left|\left|x_{i}-x_{j}\right|\right|}^{2}}{2\sigma_{i}^{2}}\right)}$$
  where $\sigma_{i}$ is the Gaussian variance centered on the data point $x_{i}$
- The second step: For the low-dimensional corresponding points $y_{i}$ and $y_{j}$ of the high-dimensional data points $x_{i}$ and $x_{j}$, a similar conditional probability can be calculated as shown in Equation (2):
  (2) $$q_{j|i}=\frac{\exp\left(-{\left|\left|y_{i}-y_{j}\right|\right|}^{2}\right)}{\sum_{k\neq i}\exp\left(-{\left|\left|y_{i}-y_{j}\right|\right|}^{2}\right)}$$
- The third step: minimize the difference between $p_{j|i}$ and $q_{j|i}$, and use the gradient descent method to minimize the Kullback-Leibler Divergence (KL-Divergence) distance [31]. The cost function uses a heavy-tailed distribution to alleviate congestion problems and SNE optimization problems.

#### 3.3.3. Kinematic Fragment Clustering

A road segment may contain different traffic characteristics, but a short trip contains fairly specific traffic characteristics. In order to meet the characteristics of China’s mixed traffic, different short trips need to be classified according to the road traffic characteristics. Short trips with the same traffic characteristics are grouped into one category, and segments of different categories are combined into typical representative conditions. Therefore, it is necessary to construct a classification model to classify different short strokes according to driving conditions (using characteristic parameter representations).

Clustering is a commonly used classification method in data analysis. It is a method of grouping samples according to data similarity without given a classification category. Because the category of each short trip is unknown, the short trip is classified using an unsupervised clustering method. Commonly used clustering methods mainly include classification (split) method, analytic hierarchy process, density-based method, grid-based method and model-based method. In addition, there are semi-supervised, spectral clustering, graph clustering and other clustering methods.

## 4. Multi-Clustering Algorithm

In this section, we will introduce in detail the structure and actual implementation steps of the multi-clustering algorithm proposed by us, explain the advantages and feasibility of the model from a theoretical perspective, and provide theoretical support for subsequent experiments.

### 4.1. Model Construction

Considering that, as mentioned above, the use of a model alone cannot achieve a good clustering effect, and will be affected by the skew of the data set distribution, making the classification inaccurate. Therefore, we consider designing a ensemble model to further improve the clustering effect of a single model based on the principle of using integrated learning. The model construction of unsupervised ensemble learning is mainly considered from the following two perspectives:

- Multi-Dataset: Drawing on the idea of bagging algorithm, we need to divide the kinematics dataset of the whole vehicle into multiple sub-datasets for clustering. However, if the sub-data sets are randomly selected in all kinematic segments directly, the kinematic segment categories in each sub-data set are unevenly distributed, and the clustering results are abnormal. Therefore, we adopted a method of randomly extracting sub-data sets according to the average speed.
- Multi-model: Based on the idea of boosting algorithm, the clustering results of the two single models described above are fused in a certain way (such as voting method, weighted average method, etc.) when we get the clustering results of multiple data sets on multiple models.

In this regard, we need to consider how to fuse multiple clustering results. We have adopted the following two options.

- Directly integrate the drawings obtained by each model.
- Drawing on the idea of stacking algorithm, the kinematic segments of each model with the most typical features are grouped into a new set of kinematics segments, and then clustered again to obtain the merged work images.

**Figure 3:**
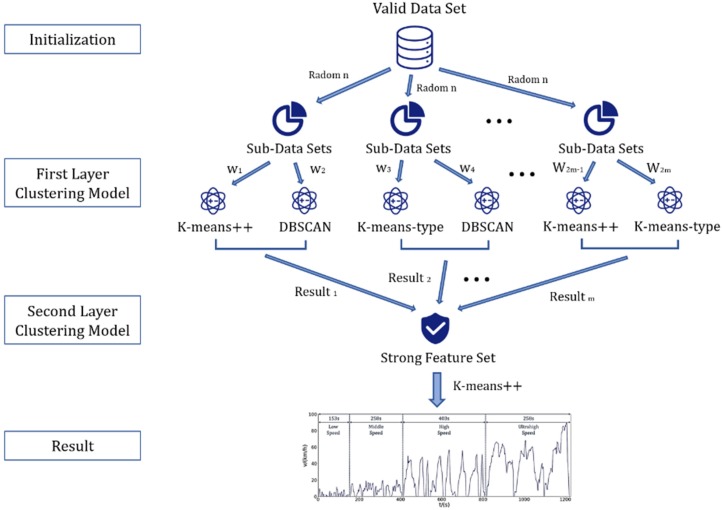
The multi-clustering algorithm framework diagram.

Among them, the first model fusion method is to first match the corresponding points of the driving cycle map obtained by each model, and then perform a weighted average and other fusion operations. Although this method is intuitive and effective, it is found in the actual operation that even if the driving cycle maps obtained by different models are not significantly different from the perspective of the overall feature vector, the corresponding points tend to be different or even have many phase offsets (one working condition). The acceleration segment of the graph corresponds to another condition map and becomes the deceleration segment. Thus, direct weighted averaging causes problems in the trend of each small kinematic segment of the drawing. Therefore, this paper uses the second model fusion method to build multi-clustering algorithm. The proposed multi-clustering overall model framework is shown in Figure 3.

### 4.2. Algorithm Implementation

We first give the detailed steps of the overall multi-clustering algorithm, as Algorithm 1 shows:

- Classify according to the average speed of short-stroke, and then randomly extract sub-data sets. Short-stroke is divided into three categories with average speeds below 10 km/h, above 70 km/h, and between the two. The number of fragments in the first two is significantly less than the third. Each time a sub-data set is extracted, it is extracted at a ratio of 1:1:2 so that the distribution of the sub-data set is relatively even. Each sub-data set is then clustered separately using two models to obtain the results.
- Select a strong feature dataset. For the clustering results of each model, the correlation analysis method is used to select the most representative kinematic fragments in each category.
- Cluster the strong feature dataset again. The data set has obvious data characteristics (small intra-class differences and large inter-class differences), so the effect of clustering will be clearer. Thereby, the final clustering result is obtained.

Then, we present the correlation analysis algorithm used in the algorithm, as Algorithm 2 shows.

Through Algorithm 1, we can obtain short strokes with strong features that better characterize the driving characteristics of the current city. The driving cycle map obtained by clustering and splicing through strong feature segments will be more representative than the results obtained by analyzing all the data. A comparison of the specific algorithm effects will be given in the next section.

## 5. Experiments

In this section, we will detail the actual data analysis, processing steps, and results and visualize and display indicators and results, and compare the different benefits and effects of different algorithms. The optimal algorithm is chosen as the basis for the classification and aggregation of short stroke segments.
**Algorithm 1:** Multi-Clustering Algorithm**Input:**  All short-stroke clips. The short-stroke segment is written as $X=\left\{x_{1},\;x_{2},\;x_{3},\;\cdots ,\;x_{t}\right\}$.  Where t is the total number of short-stroke segments. X represents the characteristic parameter $P_{i}=\left\{T_{i},\;T_{ai},\;T_{di},\;\cdots ,\;RPA_{i},\;R_{maxi},\;\cdots ,\;I_{mini},\;I_{mi}\right\}$ of the short-stroke segment. **Output:**  Strong Feature Set Y. 1: Sub−$\mathrm{data}\;\mathrm{sets}\;X=\left\{X_{1}^{\prime },{\;X}_{2}^{\prime },{\;X}_{3}^{\prime },\;\cdots ,{\;X}_{m}^{\prime }\right\}$
←Initialize_random(n) 2: **for**
$x\in X1^{\prime },X4^{\prime },\ldots ,\;X^{\prime }3p+1\;\;\;\;\;p\in N$
**do** 3:  K−means++(x, k=4), DBSCAN(x,eps) 4:  $Final\_score\leftarrow Cor\_score\left(K-means++,\;x\right)\times w_{6p+1}+Cor\_score\left(DBSCAN,\;x\right)\times w_{6p+2}$ 5:  (Initialize) Temp{} ← Rank_down(Final_score(x)) 6:  $Y_{3p+1}=\left\{\mathrm{Temp}\left[0],\;\mathrm{Temp}[1],\;\cdots ,\;\mathrm{Temp}[0.8n\right]\right\}$ 7: **end for** 8: **for**
$x\in X2^{\prime },X5\prime ,\ldots ,\;X\prime 3p+2$
**do** 9:  K−means−type(x, k=4), DBSCAN(x,eps) 10: $Final\_score\leftarrow Cor\_score\left(K-means-type,\;x\right)\times w_{6p+3}+Cor\_score\left(DBSCAN,\;x\right)\times w_{6p+4}$ 11: Temp{} ← Rank_down(Final_score(x)) 12: $Y_{3p+2}=\left\{\mathrm{Temp}\left[0],\;\mathrm{Temp}[1],\;\cdots ,\;\mathrm{Temp}[0.8n\right]\right\}$ 13: **end for** 14: **for**
$x\in X3^{\prime },X6\prime ,\ldots ,\;X\prime 3p$
**do** 15: K−means++(x, k=4), k−means−type(x,k=4) 16: $Fin\_score\leftarrow Cor\_score\left(K-means++,\;x\right)\times w_{6p+5}+Cor\_score\left(k-means-type,\;x\right)\times w_{6p}$ 17: Temp{} ← Rank_down(Final_score(x)) 18: $Y_{3p}=\left\{\mathrm{Temp}\left[0],\;\mathrm{Temp}[1],\;\cdots ,\;\mathrm{Temp}[0.8n\right]\right\}$ 19: $Y\;\leftarrow \left\{Y_{3p},\;Y_{3p+1},\;Y_{3p+2}\right\}$ 20: **end for**

**Algorithm 2:** Correlation analyze Algorithm**Input:**  The result of different cluster algorithm Y′. Each short-stroke segment has a characteristic parameter $P_{i}=\left\{T_{i},\;T_{ai},\;T_{di},\;\cdots ,\;RPA_{i},\;R_{maxi},\;\cdots ,\;I_{mini},\;I_{mi}\right\}$.  Each cluster center point parameter $Q_{j}=\left\{T_{j},\;T_{aj},\;T_{dj},\;\cdots ,\;RPA_{j},\;R_{maxj},\;\cdots ,\;I_{minj},\;I_{mj}\right\}$. **Output:**  Cor_score(P, Q). 1: **for**y∈Y**do** 2:  $Cov\left(P_{i},\;Q_{j}\right)=E\left[\left(P_{i}-E\left[P_{i}\right]\right)\left(Q_{j}-E\left[Q_{j}\right]\right)\right]=E\left[P_{i}Q_{j}\right]-E\left[P_{i}\right]E\left[Q_{j}\right]$ 3:  $Var\left(T\right)=E\{{\left[T-E\left(t\right)\right]}^{2}\}$ 4:  $\mathrm{Correlation}\_\mathrm{Score}\left(\mathrm{Qj},\mathrm{Pi}\right)=\frac{cov\left(P_{i},Q_{j}\right)}{\sqrt{Var\left(P_{i}\right)Var\left(Q_{j}\right)}}=\frac{cov\left(P_{i},Q_{j}\right)}{\sigma_{P}\times \sigma_{Q}}$ 5: **end for**

### 5.1. Data Preprocessing

In this section, we analyze and evaluate data from the perspectives of motion data processing and analysis, geographic data processing and analysis. From the perspective of actual data, it provides a reasonable basis and methodology for subsequent algorithms. Among them, the sports data mainly includes related information of motor sports, such as vehicle motion data record completion, idle duration analysis, kinematic segmentation, etc.; geographic data mainly analyzes vehicle geographic trajectories from latitude and longitude information.

#### 5.1.1. Vehicle Motion Data Processing

We used the data of three light vehicles in Fuzhou collected for 7 days and 168 h for continuous research. The sampling interval was 1 s. The basic data statistics are shown in Table 1. After analysis, we found that in the 7-day record, the three cars had data losses due to equipment failure, GPS signal disappearance, etc., and the amount of missing data was not equal. The missing amount of the vehicle 1 is the smallest, and the missing amount of the vehicle 2 is the largest. The specific missing information is shown in Table 1. For this phenomenon, we use the interpolation method for data completion, and the interpolation method is calculated according to Equations (3) and (4):

(3) $$k=\frac{b_{x}-b_{y}}{n+1}$$

(4) $$a\left(i\right)=b_{x}+k\times i$$

where $b_{x}$ represents the last value before the missing segment. $B_{y}$ represents the first value after the missing segment. K represents the slope. a(i) is the insertion value at the $i^{th}$ of the missing position.

**Figure 4:**
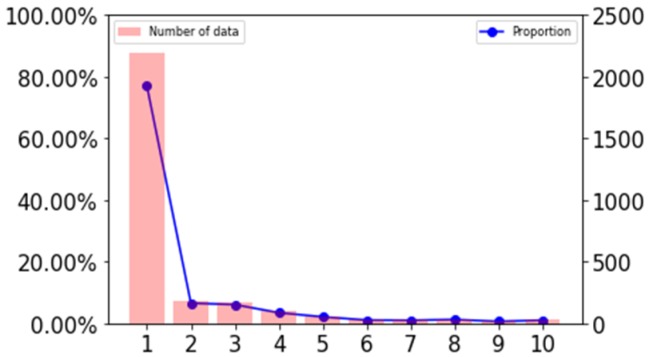
Missing duration distribution statistics.

After statistics, as shown in Figure 4, more than 70% of the missing points are less than 10s in length, so we have completed the missing fragments below 10 s. The number of records after completion is shown in Table 1.

**Figure 5:**
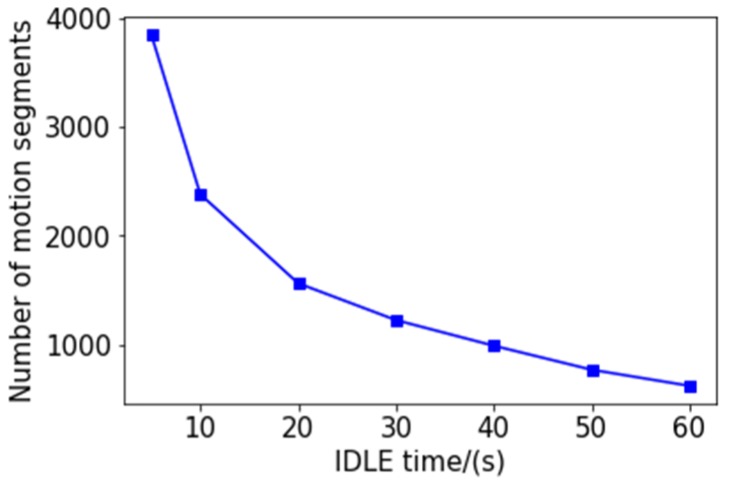
The relationship between the number of segments and the duration of continuous idle speed.

**Figure 6:**
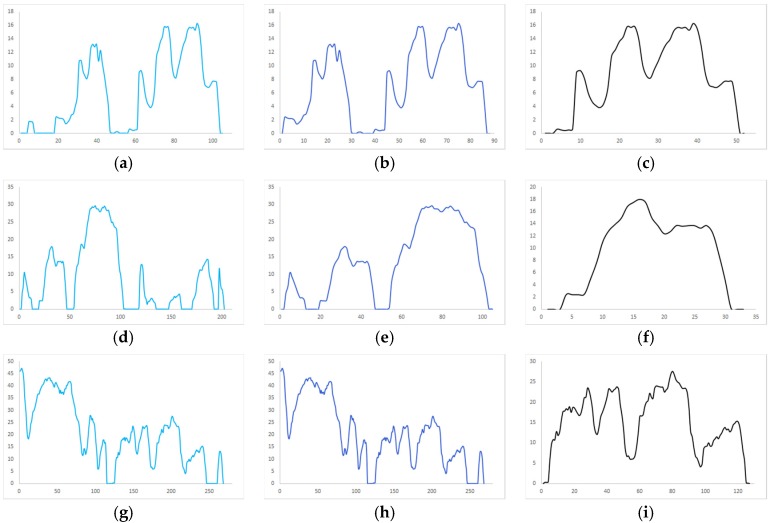
Three cases of dividing kinematic fragments according to different idle durations: (**a**) Car 1 divided into 20s; (**b**) Car 1 divided into 10s; (**c**) Car 1 divided into 5s; (**d**) Car 2 divided into 20s; (**e**) Car 2 divided into 10s; (**f**) Car 2 divided into 5s; (**g**) Car 3 divided into 20s; (**h**) Car 3 divided into 10s; (**i**) Car 3 divided into 5s.

After completing the record, we segmented according to the continuous idle duration. We try to segment in 5, 10, 20, 30, 40, 50, and 60 s, and the number of segments is shown in Figure 5. We then select three kinematic speed- time image records separately, and divide them according to different continuous idle durations, and get 9 images as shown in Figure 6.

The three maps in each row are the speed-time images of the same segment of the travel record divided by different division rules. Figure 6a,d,g are divided into continuous idle speeds of 20 s. In Figure 6b,e,h the pictures are divided into 10 s at continuous idle speed, and in Figure 6c,f,i the data is divided into 5 s segments according to the continuous idle speed. According to the continuous idle time of 20 s, a kinematic segment with a duration of several hundred seconds can be obtained, and a kinematic segment with a duration of several tens of seconds can be obtained by dividing by 5 s. Of course, the length of the kinematics segment is related to the speed of the vehicle and the road conditions. In general, we can see that the kinematics segmentation according to the continuous idle duration of 5 s can obtain a complete and independent velocity-time curve with representative features, which will greatly facilitate the accurate operation of the subsequent clustering algorithm. Therefore, we choose to divide the kinematics according to the continuous idle time of 5 s. The length and quantity distribution of the kinematics after the division are shown in Figure 6. The number of kinematic segments after segmentation is shown in Table 1.

#### 5.1.2. Geographic Data Processing

**Figure 7:**
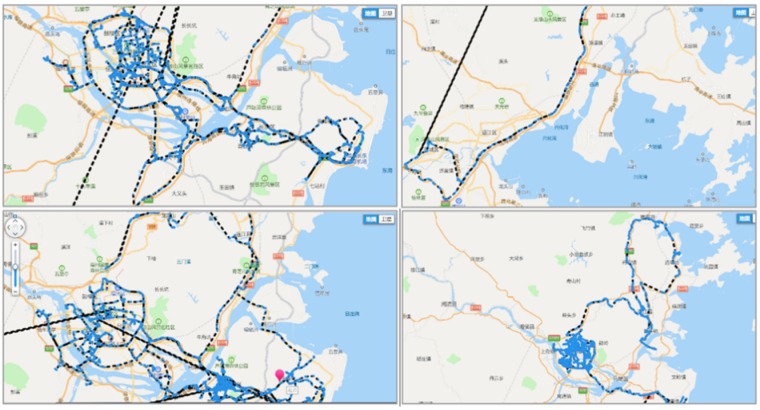
Captured track information display.

After combining the time information of the data set with the latitude and longitude information, we use the Tencent Map API to draw the motion track information as shown in Figure 7. As can be seen from the figure that the three vehicles providing the data set have driving records on urban roads and expressways, and the number of miles driven on each road is basically the same. However, the speed on the city road is slow, so it takes a long time.

### 5.2. Feature Selection and Processing

In the field of machine learning, features are the only way for machines to obtain information. Good feature selection, construction, and processing can greatly improve the accuracy of machine judgment. In this section, we will show you how to handle features in a dataset.

#### 5.2.1. Feature Building

In the study of vehicle operating conditions, it is very important to extract effective features as learning and clustering parameters. We designed and constructed 36 digital features based on the characteristics of the data set and the features commonly used in driving cycle prediction. Parameters are shown in Table 2. Among the 36 parameters we designed, there are five time-related parameters, 14 speeds, acceleration-related parameters, 10 vehicle stress situations and power-related physical parameters. These parameters can more comprehensively represent the vehicle’s operating conditions, vehicle driving conditions, and help with subsequent feature processing.

#### 5.2.2. Feature Processing

The similarity calculation formula (Euclidean distance, cosine distance, etc.) of the clustering algorithm is affected by the dimension and abnormal data. Therefore, we need to correct the outliers and normalize them. In our study, the abnormal point is corrected using the long lower bound, as shown in Equation (5):

(5) $$x=\{\begin{array}{c} x-3\delta \;\;\;\;\;(x-u<3\delta )\\ x+3\delta \;\;\;\;\;(x+u<3\delta ) \end{array}$$

**Figure 8:**
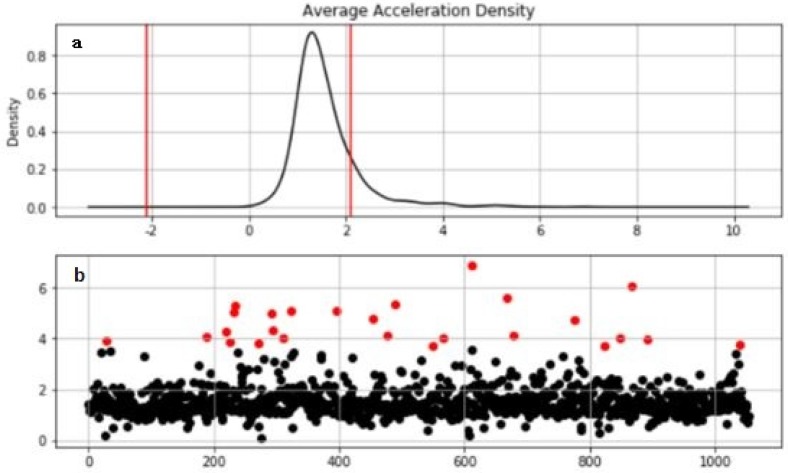
Abnormal point correction: (**a**) Acceleration distribution map; (**b**) Abnormal point.

Taking the acceleration density distribution as an example, as shown in Figure 8, the Figure 8a is the acceleration distribution map. The red point in the Figure 8b is the abnormal point and needs to be corrected.

**Figure 9:**
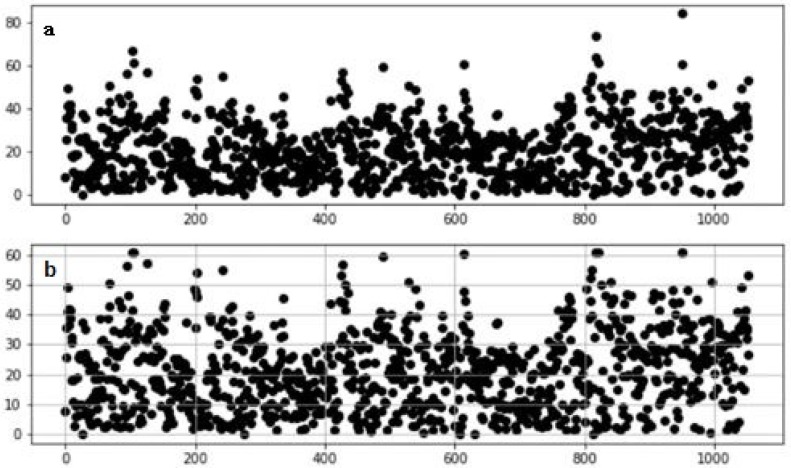
Abnormal point correction: (**a**) Comparison results before; (**b**) Comparison results after.

Figure 9 shows the comparison results before and after the correction of the outliers. As can be seen from the figure, the distribution of the corrected data is more uniform over the interval. If the outliers are not processed, normalization will cause most of the data to be compressed into a small interval, causing the distance formula to fail.

### 5.3. Feature Dimension Reduction

Since we have 36 features for the kinematics segment, the dimensions are high and some of them have redundancy features. In order to improve the efficiency of our algorithm and more accurately reflect the important features of kinematics, we need to use algorithms to reduce dimensionality of high-dimensional features. The PCA algorithm is a linear dimensionality reduction algorithm. Although PCA works well in many areas, in some complex areas, such as complex polynomial relations, nonlinear transformation methods are needed to reduce dimensionality.

First, we use the Python *sklearn.decomposition* library to analyze the contribution rate and cumulative contribution rate of the 36 features proposed in the previous section. Obtain reliable indicators of dimensionality reduction from the perspective of data analysis. It is generally believed that when the cumulative contribution rate of the principal component exceeds 85%, this part of the principal component contains most of the information in the original indicator.

Through statistical analysis, we obtain the principal component contribution rate and the cumulative contribution rate as shown in Table 3 (arranged in descending order). As can be seen from Table 3, the cumulative contribution rate of the components $M_{1}$, $M_{2}$, and $M_{3}$ has reached 85%, so we choose to keep the three components of $M_{1}$, $M_{2}$, and $M_{3}$, and reduce the dimension to 3 dimensions. Then we used the T-SNE and PCA algorithms to try in the vehicle 1 dataset. The comparison proves that the T-SNE algorithm performs better on our dataset.

**Figure 10:**
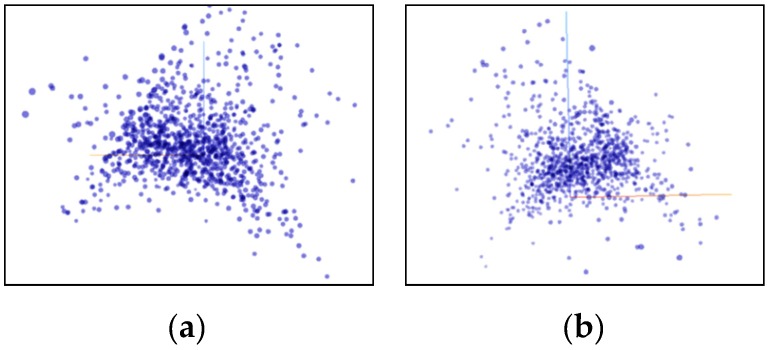
Use PCA to reduce the dimension to a 3D rendering. (**a**) x-y plane viewing angle; (**b**) y-z plane viewing angle.

**Figure 11:**
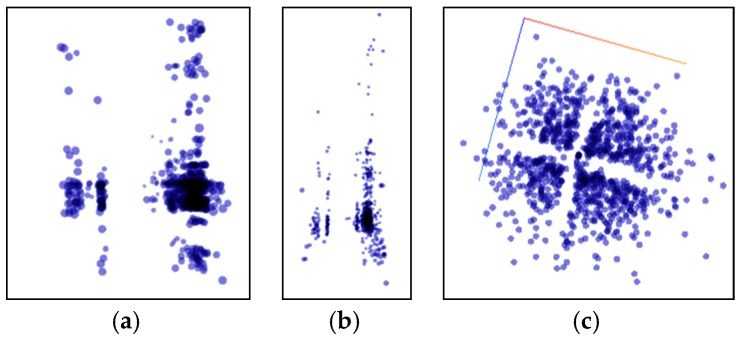
Use the T-SNE algorithm to reduce the dimensional effect. (**a**) Iteration 100 times; (**b**) Iteration 300 times; (**c**) Iteration 1000 times.

Figure 10 shows the data distribution characteristics when the high-dimensional feature of the vehicle 1 data set is reduced to three dimensions using the PCA algorithm. It can be seen from the observations of two different angles that after the dimensionality reduction using the PCA algorithm, the feature points are not grouped clearly, there are many outliers, and there is no clear classification center. Figure 11 shows the data distribution characteristics when the high-dimensional feature of the vehicle 1 data set is reduced to three dimensions using the T-SNE algorithm. The three graphs from left to right show the changes in the dimensionality reduction effect as the number of iterations increases. It can be seen from Figure 11a,b that the algorithm clearly divides the feature points into two left and right aggregates, and the similar points are close to each other, and there are clear isolation bands between the different types of points. Figure 11c is an algorithmic effect of iteration 1000 times, and the results given by the algorithm have basically stabilized. The algorithm explicitly divides the feature points into four categories, each with a relatively clear separation band, and the outliers are very few relative to the PCA algorithm results, and are within the engineering tolerance. Therefore, the T-SNE algorithm performs well in the current data set. Therefore, the T-SNE algorithm is used to reduce the dimension of the full data set.

**Figure 12:**
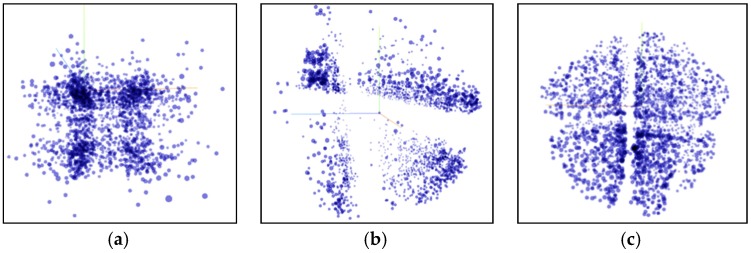
Full reduction of dimensions using the T-SNE algorithm. (**a**) Iteration 100 times; (**b**) Iteration 300 times; (**c**) Iteration 1000 times.

After individual verification, we use the T-SNE algorithm to reduce the dimensionality of high-dimensional features in the full amount of data. The characteristics after the T-SNE algorithm are shown in Figure 12. As the number of iterations increases, we can see a clear four-category state, and there is an isolation band between the classes, and the feature points within the class are close.

### 5.4. Classification and Analysis of Kinematics

In this section, we will detail how to classify and analyze kinematic segments by the features obtained after dimensionality reduction. From the perspective of practical application, compare the performance of different algorithms. And the results obtained by the algorithm with the best performance are selected as the basis for the construction of the subsequent driving condition map.

#### 5.4.1. K Value Selection

Since most clustering algorithms need to determine the number of categories, the advantages and disadvantages of the number of categories will have a greater impact on the experimental results. Therefore, based on the clustering effect, we explore the K value selection that K applies to this problem. There are two options for the selection of the number K of cluster centers in K-means:

- Make a selection based on an actual business background.
- Select based on the elbow method or contour factor, where K is selected using the elbow method. That is, the graphical tool method can be used to visualize the within-cluster sum of squared errors (SSE) according to the number of clusters. Through the graph, the influence of k on the error variance in the cluster can be visually observed to select an appropriate K value.

**Figure 13:**
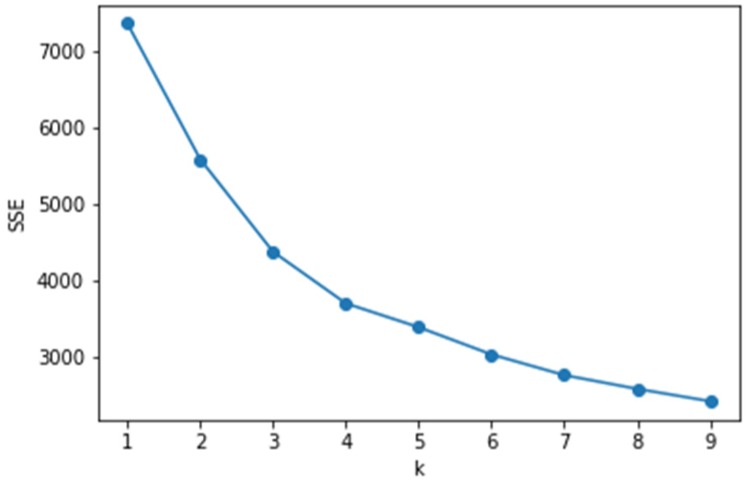
Intra-cluster error variance SSE.

It can be seen from Figure 13 that when the number of clusters is 4, the elbow type appears, which means that k is suitable. Through the analysis of the business background, the kinematics of the car can be generally divided into four states: low speed, medium speed, high speed and extremely high speed. Therefore, whether it is analysis from the business background or using the elbow method, K = 4 is a good choice.

#### 5.4.2. Clustering Results Visualization

Due to the high-dimensional features of the original features, visualization is not straightforward. Therefore, the T-SNE algorithm is used to reduce the original data and visualize the clustering results.

**Figure 14:**
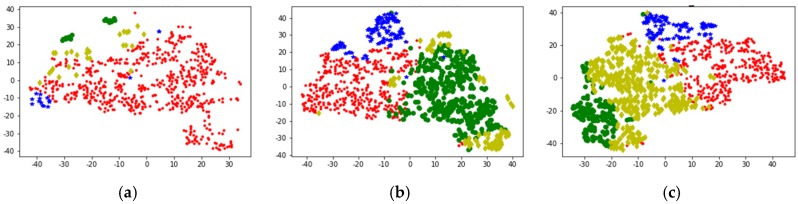
Clustering results visualization. (**a**) DBSCAN; (**b**) K-means++; (**c**) Multi-Clustering.

Compared with the K-means algorithm, the DBSCAN algorithm has the advantage of recognizing clusters of arbitrary shapes and recognizing noise. However, the number of clusters is uncontrollable, and the results of clustering are often difficult to interpret. As shown in Figure 14a, the DBSCAN results are sparse, the cluster distribution is not uniform, but some abnormal points are filtered out.

Figure 14b shows the K-means++ clustering visualization results. It can be seen that K-means++ basically divides the motion segment into four clusters. And through the visualization of the T-SNE algorithm, it can be seen that the results of the same cluster are relatively similar, but the results of the light green cluster are scattered.

Figure 14c shows the clustering effect of Multi-Clustering. It can be seen that the clustering results are divided into four categories, and the data distribution of the same cluster (same color) is concentrated, and the results of the four clusters are evenly distributed. From the results of the visualization, it is significantly better than the single model K-means++ and DBSCAN algorithms. Table 4 also validates this conclusion from the perspective of data evaluation.

## 6. Analysis of Experiments

In this section, we will perform detailed analysis based on the output of algorithms such as multi-clustering. The algorithms are quantitatively compared and conclusions are obtained.

### 6.1. Transient Driving Cycle Results and Analysis

From the engineering point of view, the construction of the driving cycle map should basically conform to the characteristic laws of the overall data, that is, the parameters should be consistent with the actual statistical measurement data. From the perspective of life, the driving cycle map is used for vehicle fuel consumption and emissions testing, and its characteristics should be consistent with the situation of the living vehicle. Vehicle condition maps that meet these characteristics are in line with reality and can be put into use. Therefore, in this section we present the driving cycle map from our two perspectives of data matching and practical application.

#### 6.1.1. Practical Application Analysis

**Figure 15:**
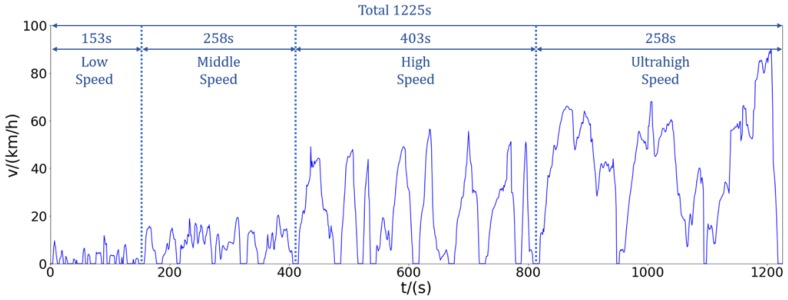
Transient driving cycle map.

The output obtained by Multi-Clustering can be obtained as shown in Figure 15. After analyzing and studying the speed, we can see from the transient condition chart that the complete speed time curve can be divided into four parts (low speed, middle speed, high speed and ultrahigh speed). From the data point of view, we can analyze that the total duration of the transient condition map is 1225 s, and the duration of each part is as shown in Table 4.

We disassemble the overall condition diagram shown in Figure 15 for analysis. Combined with the actual situation, the actual meaning of each segment of speed segmentation is explained separately. In order to analyze whether this condition map is reasonable.

**Figure 16:**
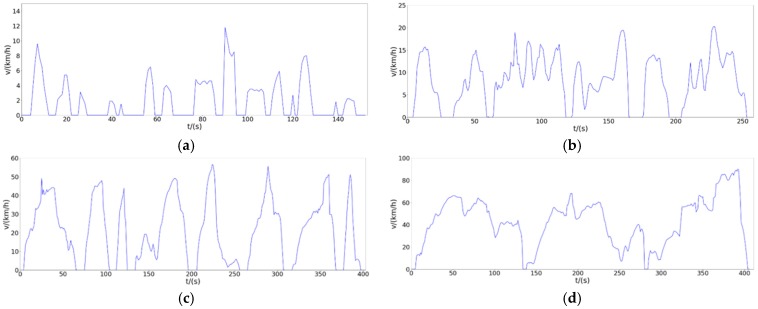
Driving cycle map detail display. (**a**) Low Speed; (**b**) Middle Speed; (**c**) High Speed; (**d**) Ultrahigh Speed.

As shown in the low-speed stage details in Figure 16a, we can see that the low-speed segment represents the speed characterization of a “crash” in life. The obvious acceleration-deceleration-parking alternation is a typical trend of daily traffic jams. As shown in the detail of the mid-speed section shown in Figure 16b, we can see that the mid-speed section represents the driving condition of the city center road during the period of heavy traffic pressure, with large traffic volume and low average speed. There is a red-light waiting time that obeys a negative exponential distribution and an acceleration/deceleration fluctuation with a minimum speed of 10 km/h. As shown in the high-speed section detail shown in Figure 16c, we can see that the section indicates the daily driving conditions of urban traffic or non-congested sections at night. The maximum speed is 60 km/h for most sections, but due to traffic lights, intersections, etc., there are frequent speed increases and decreases, and continuous driving time is not very long. As shown in the detail of the super-high-speed section shown in Figure 16d, we can see that the super-high-speed section represents the driving state of suburban roads or highways and expressways. The maximum speed is 90 km/h, the acceleration process is stable and the acceleration is high, the continuous high-speed time is long, and there is a tendency to drive for a long time. This is consistent with the analysis results of the geographic data processing part, that is, the vehicle condition map proposed by us can well match the driving situation in Fuzhou.

#### 6.1.2. Data Matching Analysis

After obtaining the working condition map, we made a detailed comparison between the proposed working condition map and the actual data set, and calculated the error rate of the measured parameters separately, as shown in Table 5. It can be seen from the table that the error rate of each parameter is within 10%. The highest error rate is the error rate of the idle time ratio, which is 9.4%, which is within the engineering tolerance. Therefore, the transient condition map we proposed is consistent with the actual data set in the data.

### 6.2. Advantages of Multi-Clustering

We use different algorithms to study the output of the worksheet for this dataset. The error rates of the results of each algorithm are shown in Table 6. We found that the error rate of the parameter map parameters and the actual data set parameters obtained by the K-means algorithm is the largest. This is due to the initial value sensitivity of the K-means algorithm, which we have already demonstrated in the algorithm analysis. Therefore, we use the improved algorithm K-means++ and DBSCAN algorithm, respectively, in the experiments. It can be seen from Table 6 that the K-means++ algorithm has low error rates such as idle time ratio, acceleration time ratio, deceleration time ratio, speed standard deviation, and acceleration standard deviation. The DBSCAN algorithm has a low error rate of average running speed, average acceleration, and average deceleration. Therefore, we try to use the integrated learning method to capture the advantages between the models, and combine the results of each algorithm to get the final vehicle condition map. As can be seen from Table 6, the error rate of each parameter in the multi-clustering algorithm results is lower than the lowest value in other algorithms. Therefore, the stacking algorithm performs well in our application.

### 6.3. Advantage Analysis of Local Cycle Map

At the beginning of this century, China directly adopted the NEDC driving conditions in Europe, and the certification of energy consumption and emissions of automobile products effectively promoted the development of energy-saving emission reduction and technology. In recent years, with the rapid growth of car ownership, China’s road traffic conditions have changed greatly. Governments, enterprises, and the public have increasingly found that cars that are calibrated and calibrated based on NEDC conditions have become increasingly biased in actual fuel consumption and regulatory certification results, affecting the government’s credibility.

The vehicle condition map we proposed is based on the data collected by Fuzhou Automobile, which is closer to China’s national conditions and is more consistent with our driving situation. We compare the working condition parameters proposed in this paper with the NEDC and WLTC working condition parameters, as shown in Table 7.

It can be seen from the table that the working conditions presented by Europe and the United States are far from our Fuzhou working conditions. The average acceleration of the NEDC condition map is significantly lower, the idle time is longer, and the acceleration time is shorter. This is because European countries generally have less road congestion. The traffic flow in Fuzhou is concentrated on the main roads, the efficiency of the distributed traffic is insufficient, and traffic congestion is prone to occur. Therefore, the situation of acceleration and deceleration is bound to be more. Thus, only the data obtained through field testing can better represent the current situation of the driving conditions of our country.

## 7. Conclusions

In this paper, we study the problem of driving cycle prediction. Two key findings found by analysis actual data are: first, the vehicle dataset used by the driving cycle prediction problem is usually unbalanced in real cases. Second, China’s geographical location and economic development are vastly different from those in Europe, not only in the various car types but also due to the complicated terrain in China. Thus, it is difficult for traditional clustering methods to achieve the effect in actual data of China. Then, we propose a novel driving cycle map algorithm framework based on ensemble learning, named multi-clustering algorithm, to improve the performance of traditional clustering algorithms on unbalanced data set. It is noteworthy the algorithm framework integrates k-means-type, K-means++, DBSCAN and other clustering algorithms, and uses stacking to strengthen each individual model. Multi-clustering can solve potential vulnerabilities such as data imbalances and random initial states. In the experimental part, we conducted multiple experiments to compare the matching order and crossover method of the algorithm to obtain the best results. It can be verified through experiments that our proposed multi-clustering framework performs well in real data sets and has a relatively high improvement over other algorithms.

Our multi-clustering algorithm is a simple and effective framework for the driving cycle prediction research. It will greatly improve the performance of traditional clustering algorithms on unbalanced data set for driving cycle prediction problems. In the future, we will continue to use the multi-clustering algorithm to examine other car parameter generation problems in complicated structure areas. We have good reason to believe that this kind of multi-clustering algorithm will play a significant guiding role in the field of unsupervised learning.

## Figures and Tables

**Table 1:** Data analysis and statistic.

	Vehicle 1	Vehicle 2	Vehicle 3
Original records	185,725	145,825	164,914
Missing fragments	724	2375	1097
After interpolation (<10 s)	185,836	147,568	165,255
Short strokes (idle > 5 s)	3849	1817	2636
Unfilled missing segments	69	130	127
Acceleration anomalies strokes	535	198	386
Short strokes too short	1190	486	1012
Final Short strokes	2055	1003	1111

**Table 2:** Vehicle driving data feature setting.

No.	Parameter	Meaning	No.	Parameter	Meaning
1	*T* (*s*)	Operation hours	19	*Rm*	Average engine speed
2	*Ta*(*s*)	Acceleration time	20	*N* *max*	Maximum torque percentage
3	*T_d_*(*s*)	Deceleration time	21	*N* *min*	Minimum torque percentage
4	*Te*(*s*)	Uniform time	22	*N* *m*	Average engine speed
5	*T_i_*(*s*)	Idle time	23	*F* *max*	Maximum instantaneous fuel consumption
6	*S*	Travel distance	24	*F* *min*	Minimum instantaneous fuel consumption
7	*V* *max*	Maximum operating speed	25	*F* *m*	Average instantaneous fuel consumption
8	*A* *max*	Maximum acceleration	26	*O* *max*	Maximum throttle opening
9	*A* *min*	Maximum deceleration	27	*O* *min*	Minimum throttle opening
10	*V* *m*	Average speed	28	*O* *m*	Average throttle opening
11	*V* *me*	Average speed of operation	29	*R* *max*	Maximum air-fuel ratio
12	*A* *a*	Acceleration section average acceleration	30	*R* *min*	Minimum air-fuel ratio
13	*A_d_*	Deceleration section average deceleration	31	*L* *max*	Maximum engine load
14	*V* *sd*	Speed standard deviation	32	*L* *min*	Minimum engine load
15	*A* *sd*	Acceleration standard deviation	33	*L* *me*	Average engine load
16	*RPA*	Relative positive acceleration	34	*I* *max*	Maximum intake air volume
17	*R* *max*	Maximum engine speed	35	*I* *min*	Minimum intake air volume
18	*R* *min*	Minimum engine speed	36	*I* *m*	Average intake air volume

**Table 3:** PCA algorithm parameter statistics.

Principal Component	Contribution Rate	Cumulative Rate
*M*_1_	65.1725	65.1725
*M*_2_	12.3442	77.5167
*M*_3_	7.8451	85.3618
*M*_4_	2.4325	87.7943
*M*_5_	1.6758	89.4701
…	…	…
*M*_36_	$1.0215\times 10^{-10}$	100

**Table 4:** Length of each speed state and its proportion.

	Low Speed	Middle Speed	High Speed	Ultrahigh Speed
Duration (*s*)	153	258	403	411
Proportion (%)	12.5%	21.1%	32.9%	33.6%
Average speed (km/h)	5.8	10.2	31.9	40.3
Maximum speed (km/h)	11.8	20.3	56.5	90.1

**Table 5:** Comparison of the driving cycle map and the actual data set parameters.

	Actual Data Set	Construction Cycle Map	Error Rate (%)
Average speed (km/h)	29.84275801	29.357573	1.6258049
Average travel speed (km/h)	26.836714	25.231541	3.9812576
Average acceleration (m/s^2^)	1.475929	1.766713	8.1835915
Average deceleration (m/s^2^)	−1.690293	−1.794614	3.7633836
Idle time ratio (%)	10.8331%	11.8544%	9.4275877%
Acceleration time ratio (%)	47.0271%	46.5026%	1.1153144%
Deceleration time ratio (%)	36.8889%	36.0751%	2.2060837%
Speed standard deviation (km/h)	19.895763	20.852563	4.8090641
Acceleration standard deviation (m/s^2^)	2.900451	2.722045	6.1509745

**Table 6:** Comparison of error rates of each algorithm.

Error Rate	Multi-Clustering	K-Means	K-Means++	DBSCAN
Average speed (km/h)	1.6258049	31.9132568	16.1388066	20.5892766
Average travel speed (km/h)	3.9812576	42.4188967	23.9006571	4.874926
Average acceleration (m/s^2^)	8.1835915	30.4949628	29.1611588	5.9235912
Average deceleration (m/s^2^)	3.7633836	26.3380964	28.5462935	6.3717231
Idle time ratio (%)	9.4275877	121.0678384	69.8147345	131.3594447
Acceleration time ratio (%)	1.1153144	14.3951041	4.878889	21.1239902
Deceleration time ratio (%)	2.2060837	6.225721	5.1630707	14.5173751
Speed standard deviation (km/h)	4.8090641	6.9892469	2.6377676	28.2313274
Acceleration standard deviation (m/s^2^)	6.1509745	5.9353873	3.2910399	14.8070421

**Table 7:** Comparison of various driving cycle map parameters.

	Actual Data Set	Fuzhou Driving Cycle	NEDC	WLTC
Average speed (km/h)	29.84275801	29.357573	33.21	22.11
Average travel speed (km/h)	1.475929	1.766713	0.540	0.392
Idle time ratio (%)	10.8331%	11.8544%	23.9%	24.2%
Acceleration time ratio (%)	47.0271%	46.5026%	22.9%	33.5%
